# De-Oiled Citrus Peels as Feedstock for the Production of Pectin Oligosaccharides and Its Effect on *Lactobacillus fermentum*, Probiotic Source

**DOI:** 10.3389/fnut.2022.826250

**Published:** 2022-05-17

**Authors:** Rohan Sarkar, Lata Nain, Aditi Kundu, Anirban Dutta, Debarup Das, Shruti Sethi, Supradip Saha

**Affiliations:** ^1^Division of Agricultural Chemicals, ICAR-Indian Agricultural Research Institute, New Delhi, India; ^2^Division of Microbiology, ICAR-Indian Agricultural Research Institute, New Delhi, India; ^3^Division of Soil Science and Agricultural Chemistry, ICAR-Indian Agricultural Research Institute, New Delhi, India; ^4^Division of Food Science and Postharvest Technology, ICAR-Indian Agricultural Research Institute, New Delhi, India

**Keywords:** pectin oligosaccharides, *Citrus* sp, probiotic activity, *lactobacillus fermentum*, XRD

## Abstract

Following the extraction of essential oil, citrus (Mousambi, Kinnow, and Orange) peel wastes were used to produce pectin. The yield of essential oil and pectin was maximum in orange. Pectin was characterized by Fourier-transform infrared spectroscopy (FT-IR) and X-ray diffraction (XRD) spectroscopy. The degree of esterification (DE) and methoxyl content (MC) was maximum in orange whereas, the equivalent weight was maximum in Mousambi. A significant increase (61.8%) in the *Lactobacillus fermentum* population was observed with pectin as compared with sugar. Three sources followed the Orange > Kinnow > Mousambi trend as a prebiotic source. It was attributed to higher DE as well as higher MC. Enhancement in the bacterial population was in the range of 79.16–87.50%. The present work confirms the potential of pectin as a probiotic source for the enhancement of the bacterial population. Thus, it has a large scope for use in the food industry targeting a circular economy.

## Introduction

With an increase in concern about consumer's health, the importance of nutraceuticals and functional foods has increased significantly across the globe. Among a number of functional foods, prebiotics find a special place, and it has attracted consumers' interest in the recent past. With the increase in the research thrust on the human microbiome, the importance of prebiotics has multiplied several times.

In this context, pectin and pectin oligosaccharides have emerged as prominent members of the prebiotic family. Structurally, pectin is a heteropolysaccharide that, remains in the primary cell walls of earthly plants. The principal component is galacturonic acid, a sugar acid derived from galactose. These heterogalacturonans are linear chains of D-galacturonic acid linked *via* α-(1–4)-linkage. Substitution over the galacturonans can also be seen where saccharide residues, such as D-apiose or D-xylose, remain attached to the backbone of residues of D-galacturonic acid in cases of apiogalacturonan or xylogalacturonan, respectively (1). Pectin is widely used in the food industry, cosmetic sector, as well as in pharmaceuticals, mainly as a gelling agent, food stabilizer, and thickener. Pectin can be acquired from a wide range of sources with a variation in yield. About 10–20% of pectin was obtained from sunflower head residues and sugar beets (2). Other sources include cocoa husk (about 9% of dry weight) (3), soya hull with 26–28% of pectin content (4), apple peel waste (1.21% on a dry weight basis) (5), apple pomace having 14–18% pectin on a dry weight basis (6). As mentioned above, citrus peels are also a potential source of pectin obtained from different species and varieties. Approximately 25% of pectin on a dry weight basis was obtained from grapefruit peels (7). A variation in yield was observed, i.e., 15–44% based on different extraction conditions and parameters from the peels of *Citrus limon* (8). Similarly, the endocarp of *Citrus depressa* has been utilized by isolating and detailed structural characterization of pectin (9).

Pectic polysaccharides help to proliferate different gut-friendly microbes as the microbial population gets increased in the course of fermentation and different short-chain fatty acids, such as acetate and propionate, are produced. This property makes these pectic substances prebiotic compounds. The prebiotic potential of pectin was evaluated using *Bifidobacterium* and *Bacteroids* by an *in-vitro* fermentation method that showed an increase in both the population of microbes (10). Pectic oligosaccharides extracted from citrus peel were tested on *Bifidobacterium bifidum* and *Lactobacillus paracasei*. An increase in cell density was observed for both the bacteria in the case of pectin compared with non-pectin substrate (11).

According to a report by the Food and Agriculture Organization out of 124.246 million tons of citrus produced all over the world, only 23.538 million tons have been utilized for processing and trade. That helps to figure out the huge waste generated from the citrus sector, mainly in the form of peels and seeds. Almost 50% of the fruit mass is under-utilized and thrown away as waste (12). But these wastes are a storehouse of various bioactive compounds having numerous health-benefitting effects, making these wastes a huge potential for utilization in various sectors commercially. Among the bioactive compounds, functional carbohydrates especially pectic substances constitute a major fraction (13).

In the field of prebiotic research, the effect of prebiotics on beneficial microorganisms especially probiotics and lactobacilli, and the selective degradation of the prebiotic by the probiotic needs more research thrust to reach a definite conclusion (14).

Considering these facts, the current experiment was designed to evaluate the yield variations of pectin from peels of three different *Citrus* sp (*Citrus limetta*, a hybrid of *Citrus nobilis* and *Citrus deliciosa*, and *Citrus sinensis*). Another objective of the study was to evaluate the effect of these pectins on the *Lactobacillus* population for their prebiotic effect to understand their potential as a potent nutraceutical product.

## Materials and Methods

### Materials

#### Peels of Citrus

Peels from three different citruses, i.e., peels of Mousambi (*Citrus limetta*), Kinnow [a hybrid between “King” (*Citrus nobilis*) and “Willow leaf” (*Citrus deliciosa*)], and Orange (*Citrus sinensis*) were taken for this study. The reason behind this is that these three types of citruses are mainly consumed in India and eventually generate more waste than others. All the fruits were collected from the local market of New Delhi, India (Azadpur fruit market; N 28.70°, E 77.10°), thereafter fruits were washed and peeled. Afterward, the peels were chopped into pieces and stored at 4°C for further use.

#### Chemicals

For extraction purposes, deionized water was utilized and obtained from a Millipore system with 18.2 MΩ cm resistance. Hydrochloric acid, ethanol, sodium chloride, sodium hydroxide, and phenol red are reagent grade and purchased from Merck® India, New Delhi. Commercially available pectin (HiMedia, Delhi) was used for the comparison of different attributes.

### Extraction of Essential Oil

The essential oil was extracted from the citrus peel by hydrodistillation using the Clevenger apparatus. Three different citrus peels (250 g) were taken in a round-bottom flask and connected with the Clevenger apparatus and condenser. Reflux the flask for 6 h to get full extraction of essential oil. After completion of the experiment, the essential oil was separated physically as well as partitioned between water and diethyl ether followed by evaporation of the solvent. The removal of traces of water was eliminated by passing through the minimum quantity of anhydrous ammonium sulfate. The yield of oil was recorded for the three peels.

#### Characterization of Essential Oil

Essential oils were characterized by gas chromatography–mass spectrometry (GC-MS) (7890A GC, Agilent Technologies equipped with an HP-5MS column (30 m × 0.25 mm × 0.25 μm, Agilent Co., CA, USA), connected to a triple-axis HED-EM 5975C mass spectrometer. Carrier gas flow was 1 ml min^−1^ and the injection volume was 1 μl. Helium was used as carrier gas at a head pressure of 10 psi. In GC, the oven temperature was initially held at 40°C for 1 min, and thereafter the temperature was raised with a gradient of 3°C min^−1^ until the temperature reached 220°C. Other settings include 250°C interface temperature and ion source temperature of 200°C.

### Extraction of Pectin

Pectin was extracted by following Ranganna's method (15). Normally bleaching is done to inactivate the enzymatic activity. Here, it was not required as it was done during hydrodistillation. Peels were kept in an oven at 60°C overnight for complete drying. After the peels were completely dried, they were ground into fine powder. About 5 g of powdered peels were put into 50 ml of 0.1 N HCl solution and stirred for 30 min at 60, 70, and 80°C. The hot extract was filtered using Whatman® Grade 1 filter paper and cooled up to 4°C. About 30 ml of ethanol was added to the extract to precipitate pectin. It was separated by filtration and freeze-dried to get pectin powder.

The percentage yield of pectin was determined using the formula,


$$\begin{array}{l} \text{Yield of pectin }\left(\%\right)=\frac{\text{Wt}.\text{ of pectin }\left(\text{g}\right)}{\text{Wt}.\text{of dried peels }\left(\text{g}\right)}\times \;100 \end{array}$$

### Physico-Chemical Characterization of Pectin

#### Moisture Content

About 1 g of pectin substance was kept for drying in an oven for 5 h at 100°C. Then, it was cooled and weighed to obtain its moisture content.


$$\begin{array}{l} \%\text{Moisture content}=\frac{\text{Final wt}.\;-\text{Initial wt}.\left(\text{g}\right)}{\text{Initial wt}.\;\left(\text{g}\right)}\times 100 \end{array}$$

#### Equivalent Weight

Pectin (0.5 g) was put in a conical flask of 250 and 5 ml of ethanol was added to it. About 1 g of sodium chloride was also added to sharpen the endpoint and 100 ml of millipore water was taken. Finally, a few drops of phenol red indicator were added followed by titration against 0.1 N NaOH. The endpoint was reached by developing a purple color. This neutralized solution was further used for methoxyl content determination.


$$\begin{array}{l} \text{Equivalent weight}\\ =\frac{\text{Wt}.\text{of sample }\left(\text{g}\right)}{\text{Volume of alkali }\left(\text{mL}\right)\times \text{Normality of alkali}}\times \;1000 \end{array}$$

#### Methoxyl (MeO) Content

Methoxy content was measured by saponification followed by titration of liberated carboxyl groups (15). The neutralized solution was taken from the equivalent weight determination and 25 ml of 0.25 N sodium hydroxide was added to it. The solution was mixed thoroughly and kept at room temperature for 30 min. After 30 min, 25 ml of 0.25 N HCl was added and titrated against 0.1 N NaOH to the same titration point as in the equivalent weight determination.


$$\begin{array}{l} \%\text{MeO}=\frac{\text{Volume of alkali }\left(\text{mL}\right)\times \text{Normality of alkali}\times 3.1\;}{\text{Wt}.\text{of sample}\left(\text{g}\right)} \end{array}$$

#### Anhydrouronic Acid (AUA) Content

The determination of anhydrouronic acid content is a prerequisite for estimating the degree of esterification and purity of pectin, using the value of the equivalent weight and methoxyl content. The AUA content of pectin was calculated by using the following formula (16):


$$\begin{array}{l} \%\text{AUA}=\frac{176\;\times 0.1\times \text{y}\times 100}{\text{w}\times 1000}+\frac{176\;\times 0.1\times \text{z}\times 100}{\text{w}\times 1000} \end{array}$$

Where 176 = molecular unit of AUA (g).

y = titer value of NaOH from equivalent weight estimation (ml).

z = titer value of NaOH from methoxyl content estimation (ml).

w = weight of sample (g).

#### Degree of Esterification

The degree of esterification (DE) of pectin was obtained based on anhydrouronic acid and methoxyl content and measured by the following formula (17),


$$\begin{array}{l} \%\text{DE}=\frac{176\;\times \;\%\text{ MeO}}{31\;\times \;\%\text{ AUA}}\times 100 \end{array}$$

Where, MeO = methoxyl content.

AUA = anhydrouronic acid content.

#### Degree of Acetylation

Pectin (0.5 g) and 25 ml of 0.1 N NaOH were mixed and stirred until the pectin was dissolved and kept overnight (15). The whole content was diluted to 250 ml by adding water and 20 ml of aliquot was taken into a distillation apparatus. Then, 20 ml of magnesium sulfate-sulfuric acid solution [magnesium sulfate (100 g) + sulfuric acid (1.5 g), diluted to 180 ml) was also put into the distillation apparatus followed by distillation. The distillate was collected at about 200 ml. This distillate was titrated with 0.05 N NaOH with a phenol red indicator. A blank distillation was also carried out with 20 ml of magnesium sulfate-sulfuric acid solution, and the distillate was titrated.


$$\begin{array}{l} \text{\%DA}\\ =\frac{\text{Volume of alkali }\left(\text{Blank}-\text{Titre}\right)\left(\text{mL}\right)\times \text{Normality of alkali }\times \;4.3\;}{\text{Wt}.\text{of sample }\left(\text{g}\right)} \end{array}$$

#### Ash Content

Pectin (2 g) was put in a crucible and heated for 6 h at 600°C under a muffle furnace (15). When the crucibles came to room temperature, they were kept in desiccators and weighed precisely. The process was repeated until a constant weight was obtained.


$$\begin{array}{l} \%\text{Ash }=\frac{\text{W}2\;-\text{W}1}{\text{W}}\times \;100 \end{array}$$

W2 = final weight of crucible and ash (g), W1 = weight of crucible, and W = weight of sample.

##### Alkalinity of Ash

About 25 ml of 0.1 N HCl was added to the ash obtained upon igniting the pectin. The mixture was then heated to a boil followed by cooling and titrated with 0.1 N NaOH using phenolphthalein as an indicator (15). A blank titration was also carried out with 25 ml of 0.1 N HCl.


$$\begin{array}{l} \text{AlkalinityofAsh}\\ =\frac{\text{Volume of alkali }\left(\text{Blank}-\text{Titre}\right)\left(\text{mL}\right)\times \text{Normality of alkali }\times \;3.1\;}{\text{Wt}.\text{of sample }\left(\text{g}\right)} \end{array}$$

### Spectroscopic Analysis of Pectin

Fourier transform infrared spectra of pectin extracted from three different sources along with commercially available pectin were recorded between 4,000 and 600 cm^−1^ on a Nicolet Nexus Avatar 370 FT-IR spectrophotometer (Nicolet, USA) having 4 cm^−1^ of resolution along with 256 scans using KBr disk as a blank (18). To understand the crystallinity of different pectin, X-ray diffraction (XRD) curves were obtained where the scanning speed was set at 1.5^0^ 2θ per min, and the scanning step of 0.1^0^ 2θ was selected during the experiment.

### Assessment of Prebiotic Effect

#### Growth Curve of Microbe Taken for Prebiotic Assessment

For this prebiotic effect evaluation of pectin, a pure culture of *Lactobacillus fermentum* was used, which was maintained by the Division of Microbiology, ICAR-IARI, New Delhi. Lactic acid bacteria were isolated from fermented food samples, collected from the native people of Himachal Pradesh, India. Isolation of *L. fermentum* bacteria through sequential methods, such as enrichment technique, acid production based qualitative screening, and followed by quantitative selection based on lactic acid production (19). The microbial strain was grown at 35°C under anaerobic conditions using de man Rogosa Sharp (MRS) broth. The growth of the microbe was monitored at regular time intervals by measuring the optical density (OD) of the medium at 622 nm.

#### Prebiotic Effect of Pectin

The response of pectin as a prebiotic was assessed by using the same culture media with a similar MRS broth composition but with the replacement of sugar with pectin. The growth of culture was also assessed in MRS broth (composition of MRS broth is given in Supplementary Table 1) with no carbohydrate source that was considered as control (20). Carbohydrate concentration was maintained at 2% in all cases. The activated inoculum was incubated with 1% (v/v) and kept at 35°C. Growth of the bacteria was observed at 12 h intervals up to 72 h when the growth of the microbe was in the stationary phase. OD values were measured as an indication of the growth of bacterial cultures (21). For further confirmation, the bacterial count was also done by taking a sample from each respective culture media by the serial dilution method using 0.9% NaCl solution.

### Statistical Analysis

Data were statistically analyzed using the open-source statistical program JASP (Version 0.14.1), and represented by using Duncan's multiple range test values. Significant differences were determined at the *p* < 0.05 level. All data were recorded in triplicate.

## Results and Discussion

### Essential Oil

With the aim of waste valorization, citrus peel was first utilized for the extraction of essential oil, and then the same de-oiled peel was dried and used for the extraction of pectin. During pectin extraction, blanching was recommended to inactivate the enzyme, which was automatically done during the hydrodistillation process. In this process, we can extract oil followed by pectin for its further utilization.

The essential oil yield was recorded for the three Citrus species and it ranged between 0.67 and 0.79% dry weight basis (Table 1). The maximum amount of the oil was reported in the Orange peel. The essential oil extracted by microwave Clevenger apparatus from Orange peel was reported to be 0.42% (22). In total, 18 Citrus species were evaluated and the average oil was reported to be 1.67% on a fresh weight basis (23). A major component of the citrus oil was limonene and it varied between 91.6 and 94.8% (Table 1, Supplementary Figure 1). Lota et al. (24) reported limonene as a major compound and it ranged between 89.1 and 95.5% across 15 mandarin species.

**Table 1:** Essential oil yield and limonene content in essential oil of three *Citrus* sp.

**Source**	**Oil content (% dry wt. basis)**	**dl-Limonene content (%area basis)**
Mosambi peels	0.74	93.17
Kinnow peels	0.67	91.56
Orange peels	0.79	94.77

### Yield of Pectin

**Figure 1:**
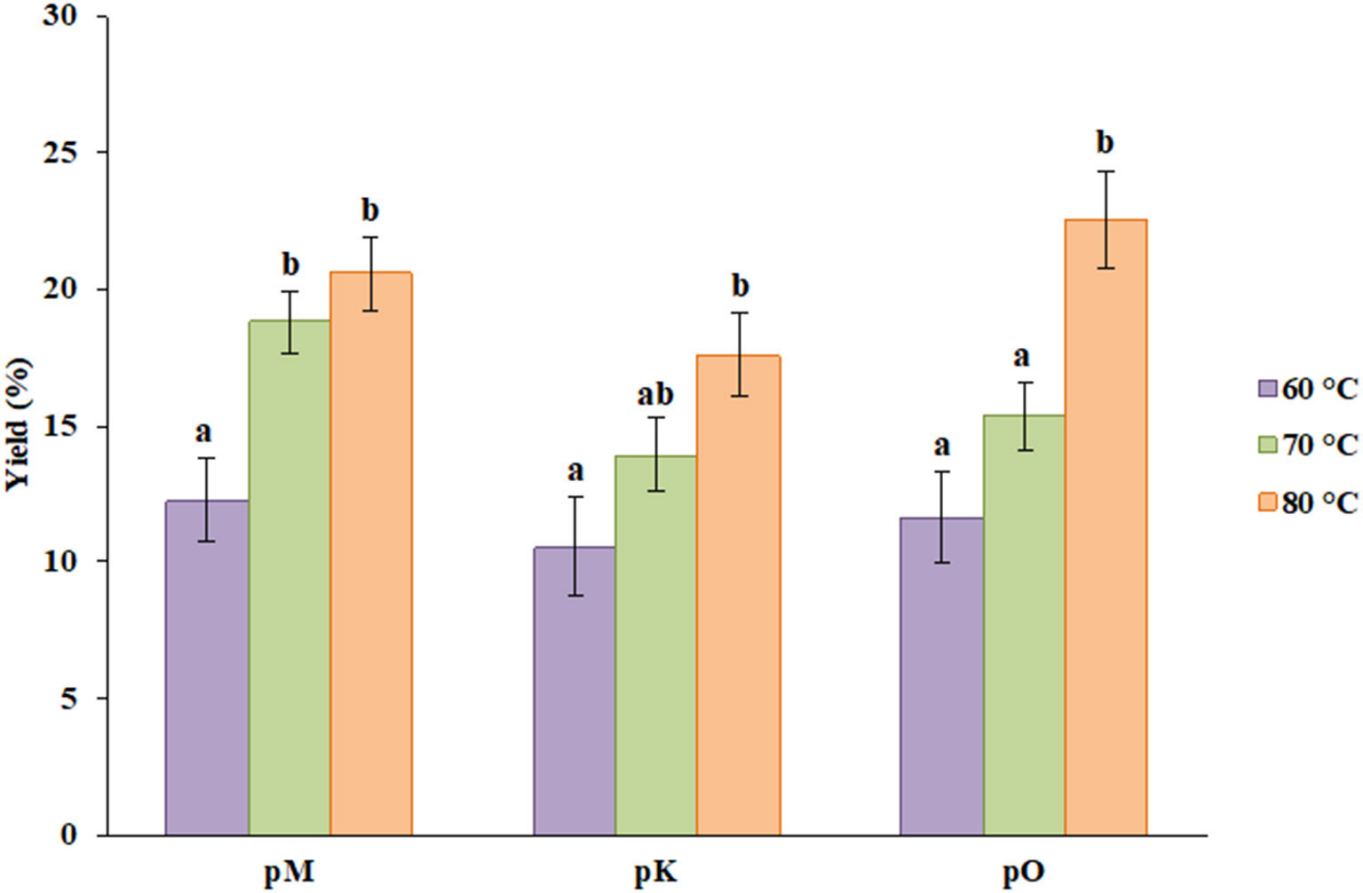
Percent yield of pectin from different sources at different temperatures where pM, pK and pO denotes pectin from Mosambi, Kinnow, and Orange, respectively. Bars sharing the same letter within a treatment are not significantly different (*P* < 0.05). Error bars represent standard deviation.

The percentage yield of pectin from three different sources was obtained at three different temperatures, i.e., 60, 70, and 80°C. As shown in Figure 1, the yield from peels of Mosambi was 12.23, 18.77, and 20.53% at particular temperatures. Similarly, 10.56, 13.89, and 17.55% yield resulted from peels of Kinnow, and 11.61, 15.32, and 22.56% yield was obtained from peels of orange. For each source, the yield has been increased by increasing temperature and the highest yield was procured at the highest temperature. This result was found to agree with Rose and Abilasha (8). As pH was fixed during the extraction procedure, the temperature played a major role in the total extraction yield. Better extraction efficiency might be attributed to better cell disruption, which was caused by higher temperature.

### Physicochemical Characterization of Pectin

Different physicochemical parameters were evaluated for the characterization of pectin from these three different sources along with commercially available pectin that has been displayed in Table 2.

**Table 2:** Physicochemical parameters of pectins extracted from three *Citrus* sp.

**Parameters**	**Pectic substances**			
	**Mosambi peels**	**Kinnow peels**	**Orange peels**	**Commercial**
Moisture%	8.19	8.24	9.26	8.08
Equivalent wt.	543.47	510.2	526.32	524.89
Methoxyl content %	6.19	6.54	6.78	7.12
Anhydrouronic acid %	67.82	71.59	71.87	73.93
Degree of Esterification %	51.81	51.86	53.55	54.67
Degree of Acetylation %	0.51	0.47	0.58	0.62
Ash %	1.34	2.12	1.02	1.78
Alkalinity of ash %	10.23	10.74	12.32	11.26

The moisture content of pectin extracted from Mosambi, Kinnow, and Orange peels was 8.19, 8.24, and 9.26%, which is slightly higher than that of commercial pectin (8.08%). Literature data about the moisture content of pectin from various citrus peels lies within 6.4–10% (25).

Equivalent weight was 543.47, 510.2, 526.32, and 524.89 for Mosambi, Kinnow, Orange, and commercial pectin, respectively, which was considerably lower than pectin from apple pomace (833.33–1,666.30) (6). The lower value of equivalent weight could denote higher partial degradation, so an increase or decrease of equivalent weight might have dependence on the presence of free acid (26). The methoxyl content of pectin is an important parameter to understand the gelling property. The values obtained were 6.19, 6.54, 6.78, and 7.12% for different pectin. Kar and Arslan (27) reported methoxyl content as 12.15% for oranges, whereas, Mohamed (7) reported approximately 7.5–8.5% of methoxyl content for grapefruit. Therefore, methoxyl content can vary with the source of material used, the method used for extraction, and other related factors. Low methoxyl pectin gels easily without the addition of sugars in the presence of divalent cations (28).

Anhydrouronic acid content varied between 67.82 and 71.87% as compared with 73.93% for commercial pectin. AUA content indicates the purity of extracted pectin. A lower value (<65%) indicates lower purity of extracted pectin with the possible presence of starch, sugars, and protein in the pectin (29). DE for all pectin was higher than 50% which also have a similarity with that of 57% (sweet orange), 56.1% (orange), and 57.1% (grapefruit) (30). As DE is >50% for the pectins understudy, they can be categorized under high ester pectins with rapid gel setting properties (31). This statement can also be argued by stating the lower values of the degree of acetylation (DA) (0.51–0.58%) for different pectin. Ranganna (15) suggested gelling capacity of pectin can be restored up to a level of 2.4% of acetyl value.

Ash content was obtained as 1.34, 2.12, and 2.12% for three different peels, similar to commercial pectin with 1.78% of ash content. Ismail et al. (29) mentioned approximately 10% of a maximum limit of ash content for good quality pectin. Ash content decreases with an increase in the yield of pectin (32). These data also agree with the statement as pectin having the lowest ash content corresponds to higher yield and vice versa. Results of percentage alkalinity of ash showed the highest value for pectin extracted from orange peels while the lowest for Mosambi peel extracted pectin. No such trend has been observed between percentage content and percentage alkalinity of ash among different pectin.

### Spectroscopic Analysis of Pectin

**Figure 2:**
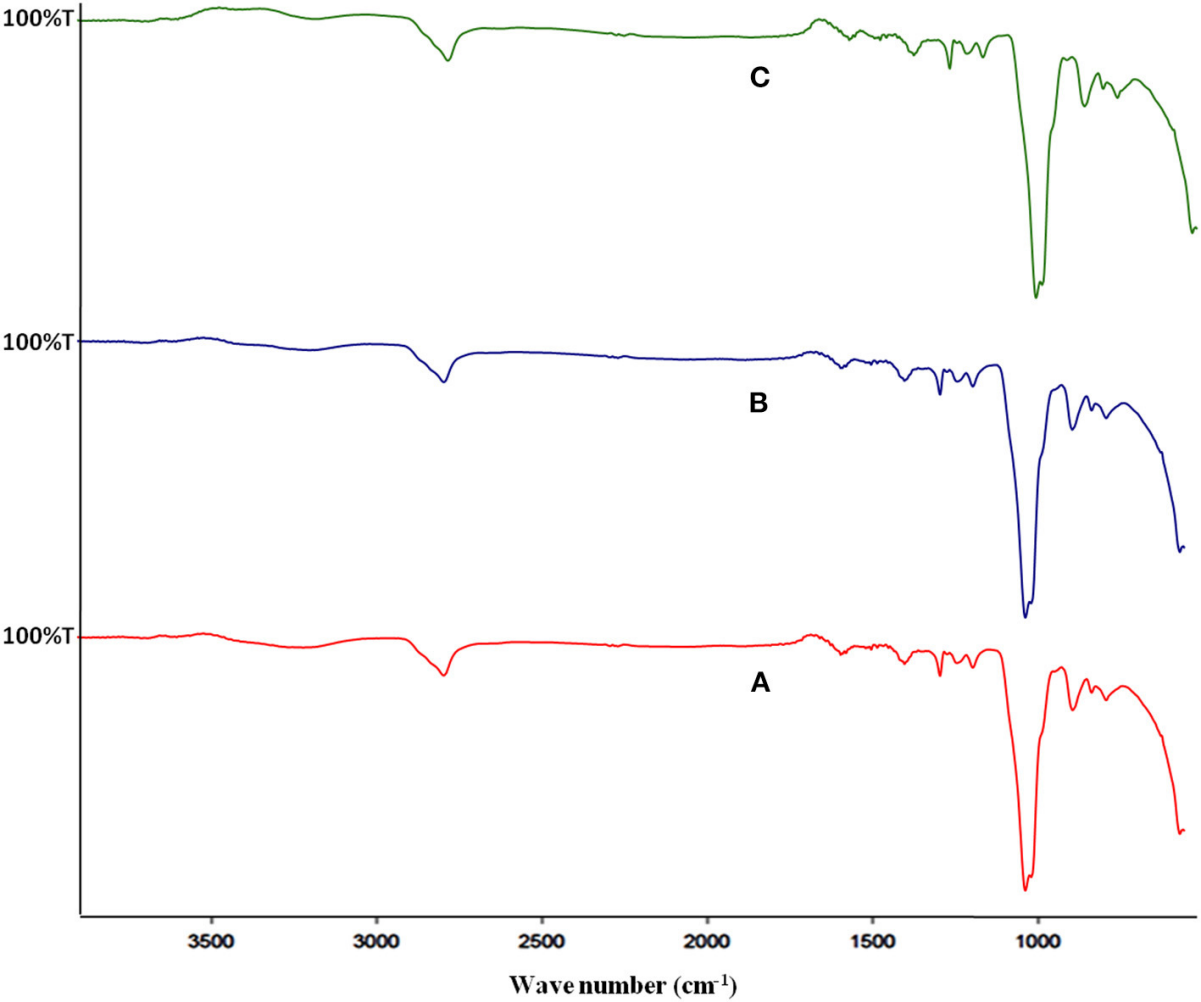
FT-IR spectra of different pectin samples, where **(A–C)** represents pectin from Mosambi, Kinnow, and Orange peels, respectively.

The FT-IR spectra of pectin samples from different sources have shown peaks at approximately 3,300, 2,900, 1,600, and 1,052 cm^−1^ that correspond to –OH stretching, –CH stretching, -C=O stretching (due to ester and acid moiety), and –COC–stretching of galactouronic acid (Figure 2). The region in the range of 3,200–3,600 cm^−1^ corresponds to the O–H stretching absorption due to inter- and intramolecular hydrogen bonding of the monomer unit of pectin. Bands (3,000–2,800 cm^−1^) are assigned to the C–H absorption that includes CH, CH_2_, and CH_3_ stretching vibrations.

In the fingerprint region (1,200–900 cm^−1^) of citrus pectin spectra, intense bands at 1,140 and 1,040 cm^−1^ were assigned to stretching vibrations of glycosidic bonds (C–O) and pyranoid rings (C–C). This showed similarity to that of IR spectra of pectin from commercial citrus pectin mixture (33, 34). The intensity of peaks corresponding to –OH stretch and =C O stretch is considerably low, which may be due to a higher concentration of sample as hydrogen bond prevails and decrease the intensity of peaks.

**Figure 3:**
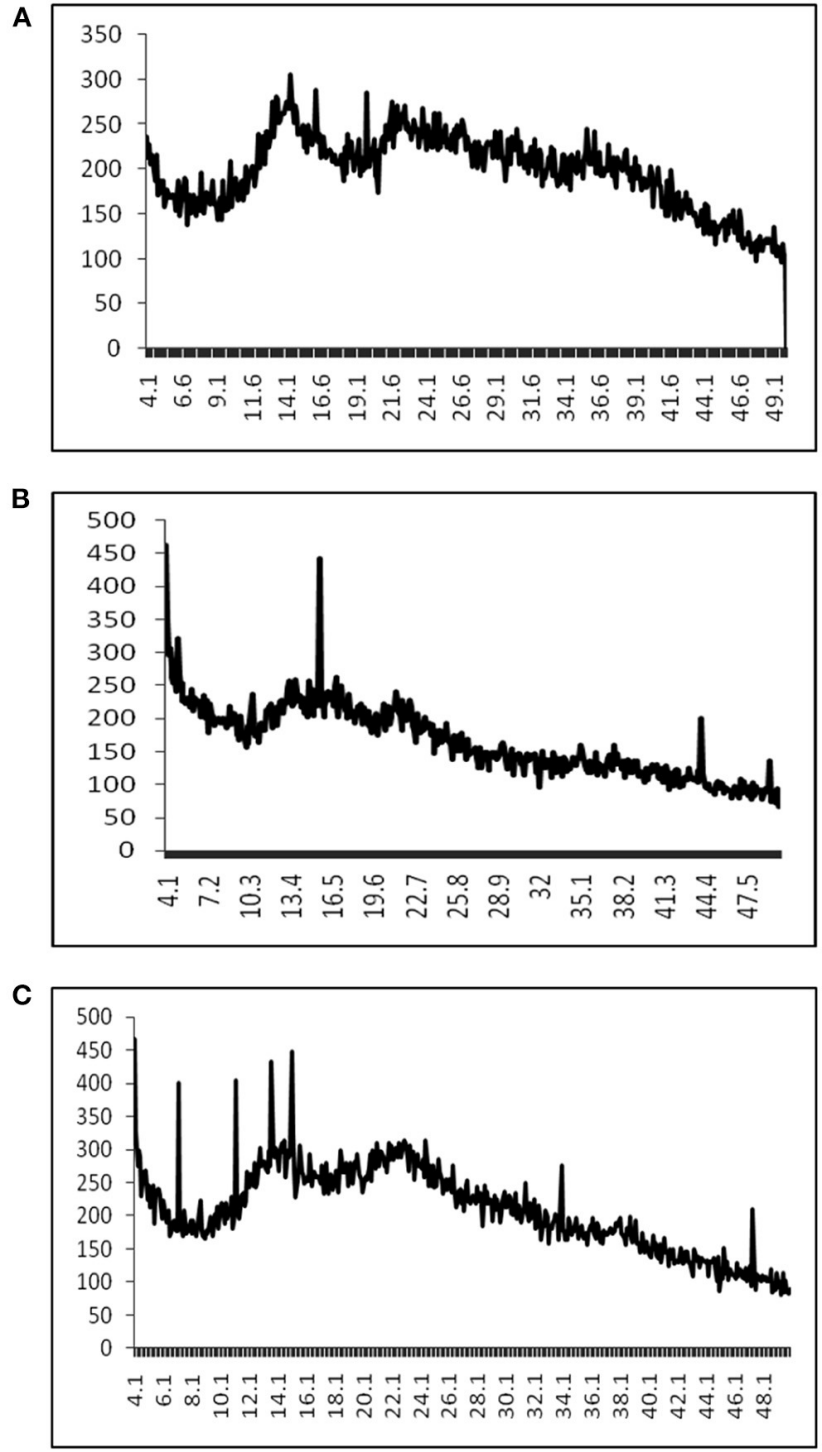
XRD of different pectin samples, where **(A–C)** represents pectin from Mosambi, Kinnow, and Orange peels, respectively.

An XRD study of different pectins showed their crystalline nature. Pectin extracted from Kinnow and Orange was more crystalline than compared with pectin from Mosambi as narrow and sharp diffraction peaks have been observed in the spectra for the former two samples but no such distinct peak was observed for latter one (Figure 3). In general, amorphous solids generally have a higher solubility and higher dissolution rate because they have higher free energy than corresponding crystals (35). The crystalline nature of pectin was also different for pectin extracted from two different varieties of apples (6) that support the fact of being different crystalline nature of pectin in this study.

### Effect of Extracted Pectin on *L. fermentum*

**Figure 4:**
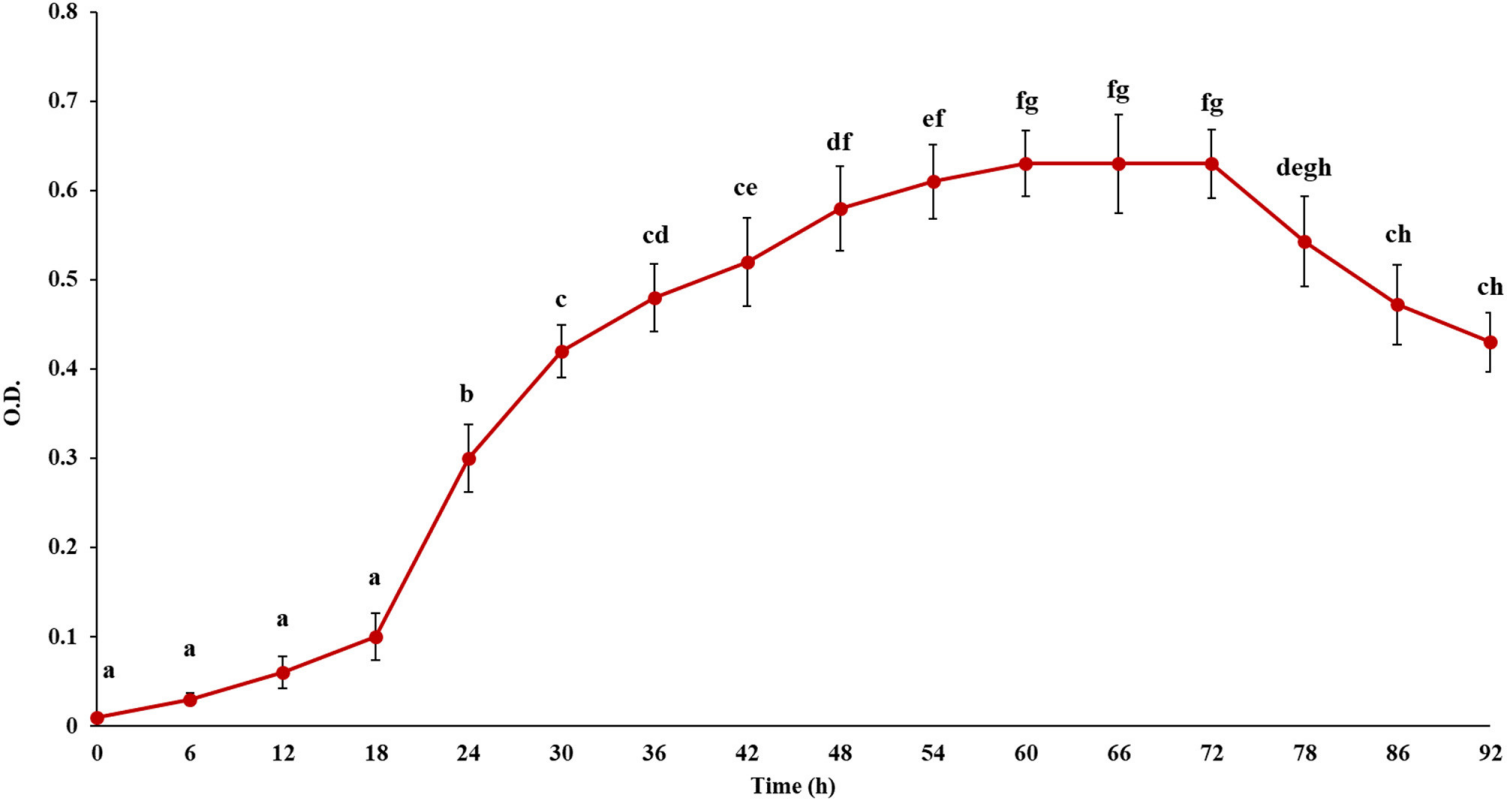
Growth curve of the probiotic, *Lactobacillus fermentum*. Points sharing the same letter are not significantly different (*P* < 0.05). Error bars represent standard deviation.

In this study, first the growth of the microbe, i.e., *L. fermentum* was observed to decide the incubation period to perform the prebiotic activity. Figure 4 depicts the change in OD in course of the growth of the microbe. It has been seen that the lag phase lasted for 18 h. Then the strain grew exponentially, which suggested it was in the logarithmic phase. After that stationary phase began at 60 h and they started to decline after 72 h. Therefore, the prebiotic activity was studied up to 72 h after culture fermentation to understand the effect of pectic substance on the microbial population.

**Figure 5:**
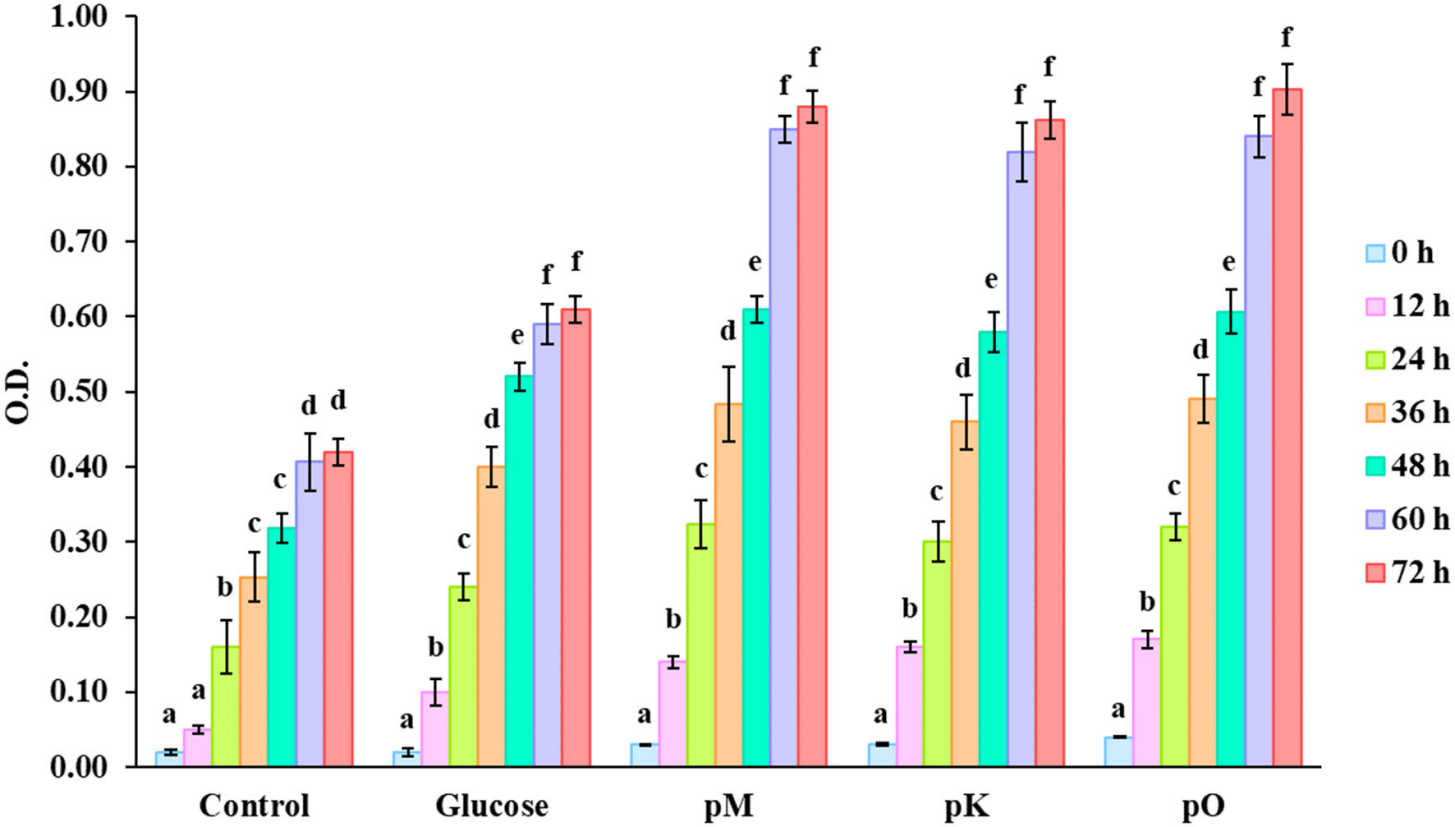
O.D values of culture media with no carbon source; control, carbon source having glucose, pectin from Mosambi peels (pM), Kinnow peels (pK) and Orange peels (pO). Bars sharing the same letter within a treatment are not significantly different (*P* < 0.05). Error bars represent standard deviation.

Figure 5 displays an increase in the OD value of culture medium fermented 72 h with pectin extracted from different citrus peels as sources of carbon. A higher OD value denotes better growth of microbe and efficient utilization of pectin. During the logarithmic phase (up to 48 h) as microbes grew rapidly, there was a minute variation between the OD value of medium added with glucose and pectin as carbon source. But in the stationary phase (up to 72 h), the OD value was distinctly higher in the case of a medium with different pectin compared with glucose as a carbon source. This type of trend indicates effective fermentation of pectin by microbe over a longer period. Tingirikari (36) studied *in-vitro* prebiotic analysis of pectic polysaccharides over various gut-friendly microbes and obtained similar kinds of findings. As these pectic substances get fermented, different organic acids are formed that help to increase the microbiome, making these substances an effective prebiotic ingredient. Furthermore, inconsistent data were reported by different authors on the ability of pectin and related oligosaccharides on the growth of specific bacterial populations (37). Decreased or unchanged level of certain bacteria, such as bifidobacteria and *Roseburia*, was reported earlier (38–40).

**Figure 6:**
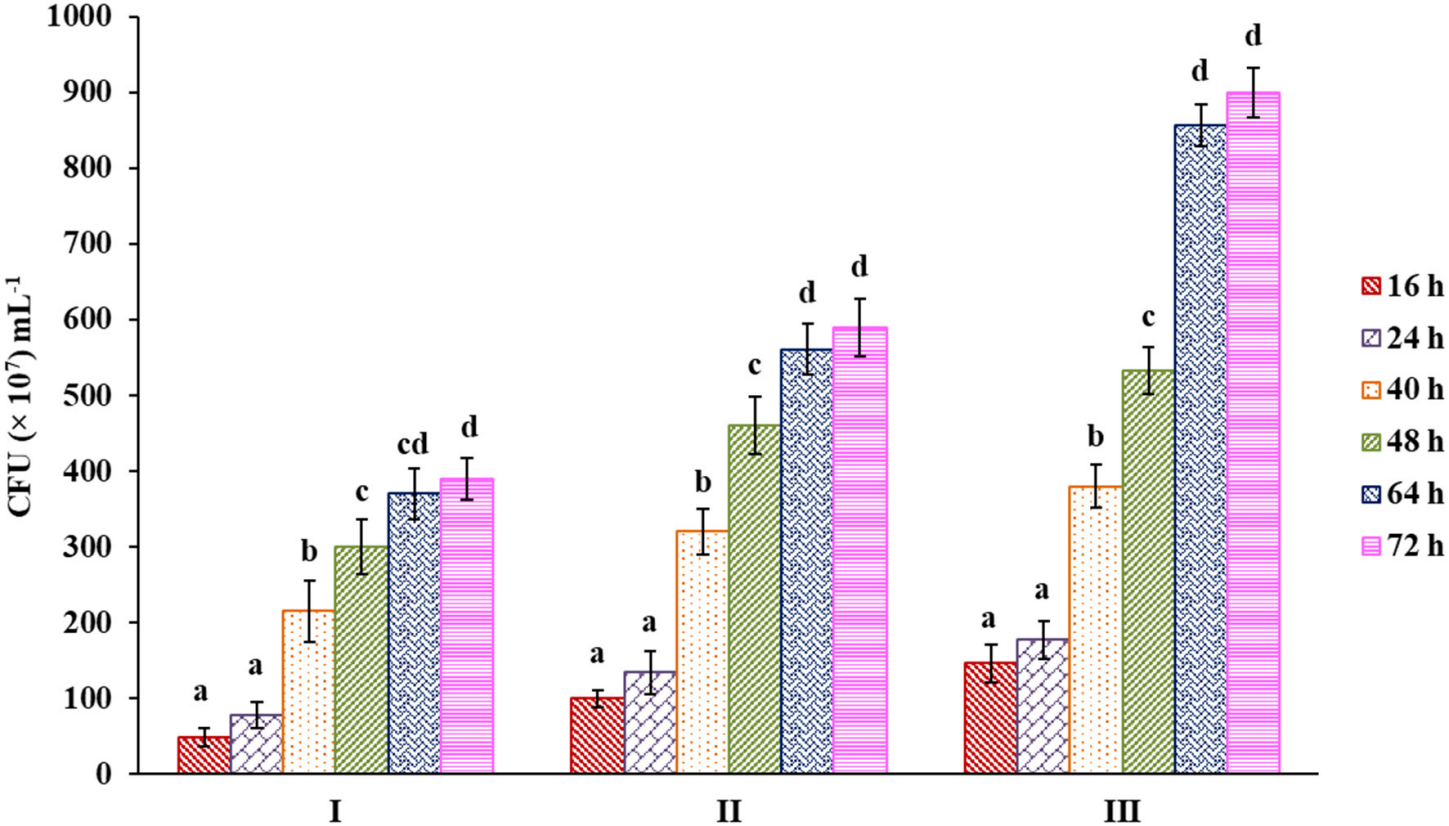
Bacterial count from samples of culture media with control (I), sugar (II) and pectin (III) as carbon source. Bars sharing the same letter within a treatment are not significantly different (*P* < 0.05). Error bars represent standard deviation.

This result was further confirmed by counting bacterial colonies from each respective culture medium having glucose and pectin as carbon sources along with the control that showed a similar kind of trend as before (Figure 6, Supplementary Figure 2). A higher bacterial population was observed in the case of pectin than in others up to 72 h, showing a nearly 61.75% increase compared with glucose as a carbon source.

### Structure-Function Relationship

Upon comparative characteristic evaluation of three different citrus peel pectin and its related prebiotic activity, the structure-activity relationship was drawn. Methoxyl content was highly correlated with the prebiotic activity. Methoxyl content followed the order pO > pK > pM and the prebiotic activity after 72 h followed a similar trend (enhancement in bacterial population was observed at 79.16, 83.33, and 87.50%, respectively). Pectin, indigestible by human enzyme, upon fermentation to short-chain fatty acids, contributes significantly to the microbiome. Production of short-chain fatty acids was higher in pectins with high methoxyl content (37). Interestingly, the DE also followed the same trend as methoxyl content. The higher the DE more the production of short-chain fatty acids and it contributed more bioactivity as prebiotics (37). The study recorded important information regarding the prebiotic potential of different pectins varied across molecular weight, structure, DE, etc. The suitability of pectin for its application is governed by the structure of pectin, including its DE, amount of methoxy, and acetyl esters (41, 42). Gomez et al. (43) reported an increase in the population of bifidobacteria and lactobacilli in the range of 19–32%.

## Conclusion

Citrus peel wastes from *C. limetta*, a hybrid between *C. nobilis* and *C. deliciosa*, and *C. sinensis* were first utilized for the extraction of essential oil, and then they were further processed for the production of pectin. Higher essential oil and pectin yield was observed in the case of orange peels than the other two species. Three different pectins were characterized for their physicochemical and spectroscopic attributes. Three pectins differ in terms of equivalent weight, DE, and methoxyl content. FT-IR spectra showed similar chemical composition but the XRD study displayed differences in crystalline nature among pectin from different sources.

The count of *Lactobacillus* sp. was enhanced significantly in the presence of pectin as compared with sugar source. Among three different pectins, enhancement of the population of the probiotic bacteria followed the order pO > pK > pM. The present study recorded significant enhancement of the probiotic bacteria, which was earlier reported as non-conclusive.

A prebiotic assay over a gut beneficial microbe notified increase in the population, which will help to enrich the gut microbiome. This piece of experiment might be helpful for the utilization of pectin in the food/nutraceutical industry as a promising product by effective use of waste materials generated regularly. Pectin can be used for the production of jams, jellies, frozen foods, and low-calorie foods as a fat and/or sugar replacer.

Citrus essential oil is being used in the food as well as cosmetic industry. So, citrus peel, which is a waste material for the juice industry, can be explored for the production of essential oil first and then for the production of pectin. Both these bioactive compounds can be successfully utilized by the food industry.

## Data Availability Statement

The original contributions presented in the study are included in the article/Supplementary Material, further inquiries can be directed to the corresponding author/s.

## Author Contributions

RS: investigation, methodology, and writing—original draft. LN: conceptualization and methodology. AK: conceptualization and formal analysis. AD: validation and resources. DD: methodology and data curation. SSe: data curation and validation. SSa: data curation, formal analysis, and writing—review and editing. All authors contributed to the article and approved the submitted version.

## Funding

The present research work was supported by the Senior Research Fellowship, Indian Council of Agricultural Research, New Delhi, India.

## Conflict of Interest

The authors declare that the research was conducted in the absence of any commercial or financial relationships that could be construed as a potential conflict of interest.

## Publisher's Note

All claims expressed in this article are solely those of the authors and do not necessarily represent those of their affiliated organizations, or those of the publisher, the editors and the reviewers. Any product that may be evaluated in this article, or claim that may be made by its manufacturer, is not guaranteed or endorsed by the publisher.
