# Supplementary material for: De-Oiled Citrus Peels as Feedstock for the Production of Pectin Oligosaccharides and Its Effect on Lactobacillus fermentum, Probiotic Source
Source: Front Nutr. 2022 May 17;9:826250. doi: 10.3389/fnut.2022.826250 (PMC9152367; doi:10.3389/fnut.2022.826250)
Supplement: Supplementary file 1 [file Data_Sheet_1.docx]

| Ingredient name | Concentration (g L^-1^) |
| --- | --- |
| Peptone proteose | 10 |
| Meat extract | 10 |
| Tween 80 | 1 |
| Yeast extract | 5 |
| Sodium acetate | 5 |
| Ammonium citrate | 2 |
| Dipotassium hydrogen phosphate | 2 |
| Magnesium sulphate | 0.2 |
| Manganese sulfate | 0.05 |
| Glucose | 20 |

Table S1. Composition of MRS broth

Fig. S1. Pictures of Bacterial plate having culture media with control (I), sugar (II) and pectin (III) as carbon source

Fig. S1. Mass spectrum of limonene as major component


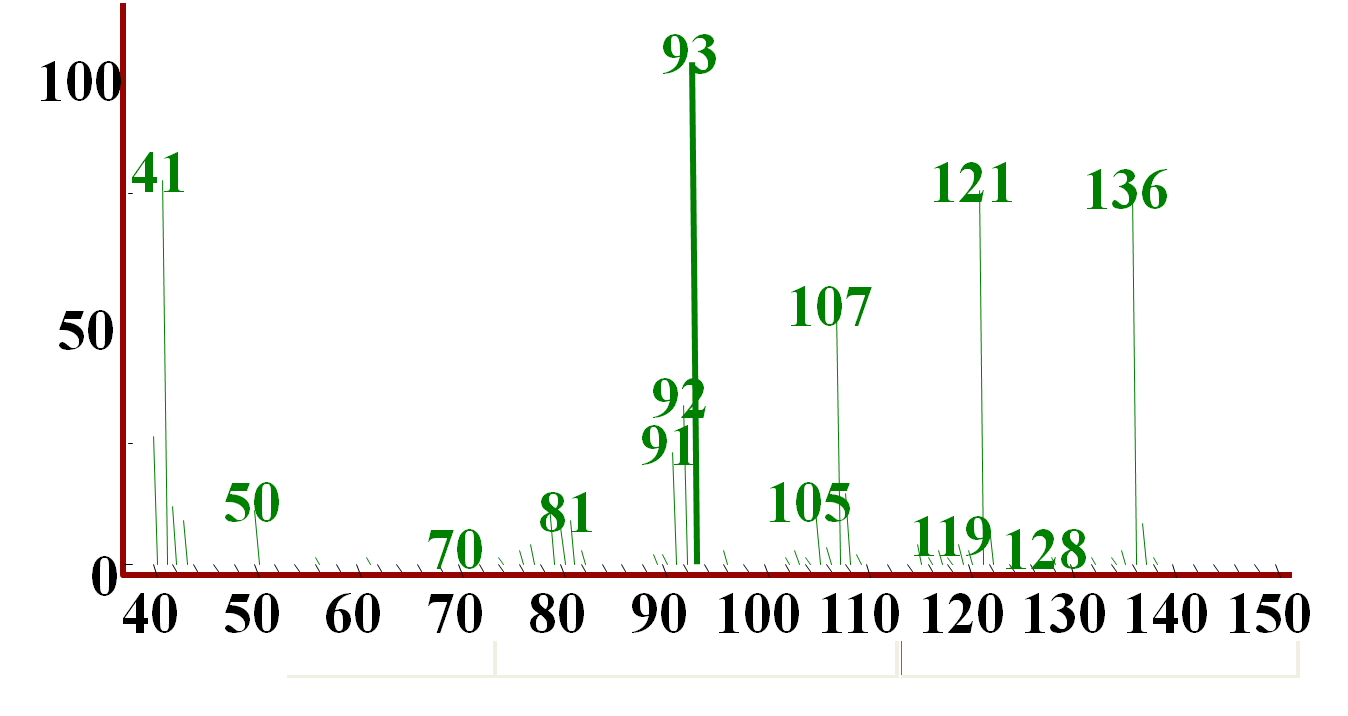


Fig. S2. (A) Bacterial plate having culture media with control (I), sugar (II) and pectin (III) as carbon source; (B) De-noised version of the picture made by using NIS Elements, Nikon using artificial intelligence


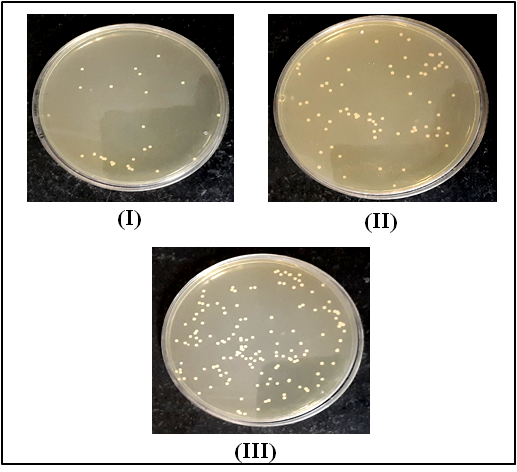
(A)


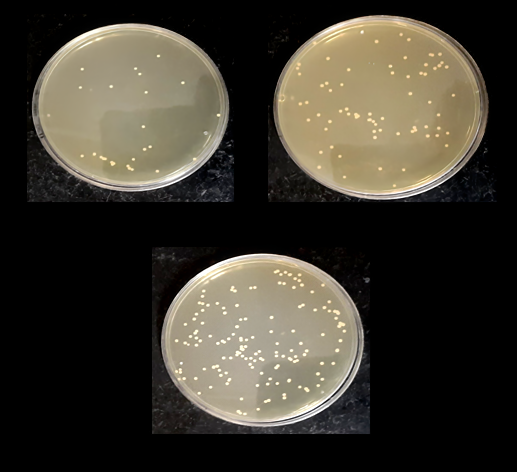
 (B)
